# Casein fibres for wound healing

**DOI:** 10.1098/rsif.2023.0166

**Published:** 2023-07-26

**Authors:** Jubair Ahmed, Ece Guler, Gul Sinemcan Ozcan, Muhammet Emin Cam, Shervanthi Homer-Vanniasinkam, Mohan Edirisinghe

**Affiliations:** ^1^ Department of Mechanical Engineering, University College London, London WC1E 7JE, UK; ^2^ Department of Pharmacology, Faculty of Pharmacy, Marmara University, Istanbul 34668, Turkey; ^3^ Center for Nanotechnology & Biomaterials Application and Research, Marmara University, Istanbul 34722, Turkey; ^4^ Stem Cell and Gene Therapies Research and Applied Center, Medical Faculty, Kocaeli University, Kocaeli 41380, Turkey; ^5^ Biomedical Engineering, Aveiro University, Aveiro 3810-193, Portugal

**Keywords:** wound healing, *in vivo*, fibres, casein, bandages

## Abstract

The name casein is given to a family of phosphoproteins which is commonly found in milk. Until recently, this was a constituent of milk that was commonly discarded; however today, it is widely used in health supplements all over the world. In this work, a high loading (50 wt%) of casein is mixed with a solution of polycaprolactone (PCL) to produce bandage-like fibres with an average fibre diameter of 1.4 ± 0.5 µm, which would be used to cover wounds in a series of tests with diabetic rats. Mouse fibroblast cell viability tests show that the casein-loaded fibres had little cytotoxicity with over 90% observed viability. A 14-day *in vivo* trial involving three groups of rats, used as control (no treatment), pure PCL fibres and casein-loaded fibres, showed that the casein within the fibres contributed to a significantly more extensive healing process. Histological analysis showed increased development of granulation tissue and follicle regrowth for the casein-loaded fibres. Further analysis showed that casein-loaded fibres have significantly lower levels of TNF-α, TGF-β IL-1β, NF-κB and IL-6, contributing to superior healing. The results presented here show an economical and simple approach to advanced wound healing.

## 1. Introduction

Biologically active peptides have gained much recent interest owing to their desirable biological properties such as being antithrombotic, antimicrobial, antioxidant, anti-inflammatory and having low toxicity [1]. In this context, these peptides can be defined as a specific portion of a protein with an amino acid length between 2 and 20 that have a desired biological effect. Many types of peptides exist that have wide use in food science and nutrition as they have been demonstrated to possess physiological roles such as the ability to enhance calcium absorption, be immunostimulating, anti-hypersensitive and even have opioid-like features [2–4]. These peptides can be sourced from a wide range of protein sources such as soy milk, rice, soy, wheat, cow's milk, rapeseed and casein [5–7].

Milk is a remarkable resource and a great supply of nutrients, vitamins, minerals, and amino acids, which also houses a wide range of biologically active peptides which have been encrypted into its proteins and can promote the development of the gut while protecting against infection [8,9]. Studies show that there are encrypted bioactive peptide sequences within casein [10]. Casein is the collective name given to a group of phosphoproteins that are prevalent in mammalian milk, with about 20–60% of human milk comprising casein, and 80% of cow's milk [11]. Its main role in the mammary gland is believed to be in preventing pathological calcification [12]. There are four subtypes of casein including *α*_S1_-casein, *α*_S2_-casein, β-casein and κ-casein, where the main fraction of milk protein consists of a mix between *α*S1- and *α*S2-casein [13].

Casein has been found to have many beneficial effects in the body. For example, many animal studies into the use of casein have shown it to be able to inhibit mutagens that cause cancer, such as dimethylhydrazine [14,15]. Casein has shown effectiveness as a biodegradable nerve guide conduit material [16]. Casein has also been linked to studies that show it is effective in preventing breast and colon cancer by the activation of signalling mechanisms which are able to suppress tumours [17]. Work has furthermore established that peptides can be encrypted within casein proteins and released during gastrointestinal digestion via proteolysis [18]. These peptides have many useful biological effects such as being antithrombotic, immunomodulatory, anti-hypersensitive and even antimicrobial [19,20]. For example, caseinomacropeptide is a biologically active peptide derived from κ-casein, which has attracted a lot of recent attention due to its many beneficial health properties. Caseinomacropeptides have the ability to inhibit bacterial adhesion by binding to *Escherichia coli* and cholera endotoxins, regulate blood circulation and even modulate immune system responses [21].

Casein and its derivatives therefore offer an important source of bioactive peptides that can be beneficial in wound healing applications through numerous ways. Casein-derived immunopeptides can stimulate the immune system (immunomodulation) by promoting the proliferation of human lymphocytes, antibody synthesis and phagocytic activities [22]. These immunoproteins are also beneficial in wound healing due to their antimicrobial potential where they could stimulate the proliferation of natural killer cells and T cells, which come into the defence against a large number of bacteria [19]. For example, immunopeptides of *α*_S1_-casein and β-casein that have been given intravenously to mice have been shown to induce phagocytosis of sheep red blood cells by murine macrophages and protect against pneumonia [23]. To date, there is little evidence in the literature which explores the *in vivo* performance and toxicity of casein-based wound healing fibrous scaffolds.

In recent years, there has been a substantial increase in the interest surrounding the production of biodegradable polymer blends which have uses in tissue engineering and wound healing [24]. Several manufacturing processes have been explored, which can fabricate such micro- or nanoscale matrices including electrospinning, phase separation, self-assembly and template-directed synthesis [25]. Pressurized gyration is another method of producing fibrous matrices from a range of different polymers and blends which has the benefit of rapidly producing masses of material, and it has also attracted much interest and publication [26–29]. This method of fibre production has importantly shown that the manufacturing of fibrous constructs for wound healing purposes is feasible, and that many natural materials with wound healing properties can be exploited [30–32]. In this work, casein-loaded polycaprolactone (PCL) fibres are spun with pressurized gyration into bandage-like constructs in order to ascertain their suitability as wound healing materials through a series of analytical techniques, which test the *in vivo* and biochemical performance.

## 2. Material and methods

### 2.1. Solution preparation

For this study, casein from bovine milk (CAS: 9000-71-9) was obtained from Scientific Laboratory Supplies (Ireland) in powder form. PCL of average Mn 80 000 (CAS: 24980-41-4) was obtained from Sigma-Aldrich (Gillingham, UK) and was used as the carrier polymer to incorporate casein into, the molecular weight being chosen to ensure that the fibres had sufficient polymer chain entanglement and could produce viable fibres [31–34]. As casein was the main active ingredient and the focus of the study, high loadings of it in solution form would be favourable; for this reason, a high weight loading of 50 wt% casein was prepared by adding casein to a 15% (w/v) solution of PCL, dissolved in chloroform (CAS: 67-66-3), obtained from Sigma-Aldrich (Gillingham, UK). The 50 wt% loading of casein equals half the weight of the PCL in the final fibre. The solutions were subjected to high-speed mixing using a DAC 150.1 FVZ-K speed mixer (DAC, Germany) for 5 min at 3500 rpm and left to dissolve over a 24-h period at ambient conditions (24°C and 35–45% relative humidity). Once dissolved, the polymer solutions would then be spun using pressured gyration.

### 2.2. Fibre generation

**Figure 1.**
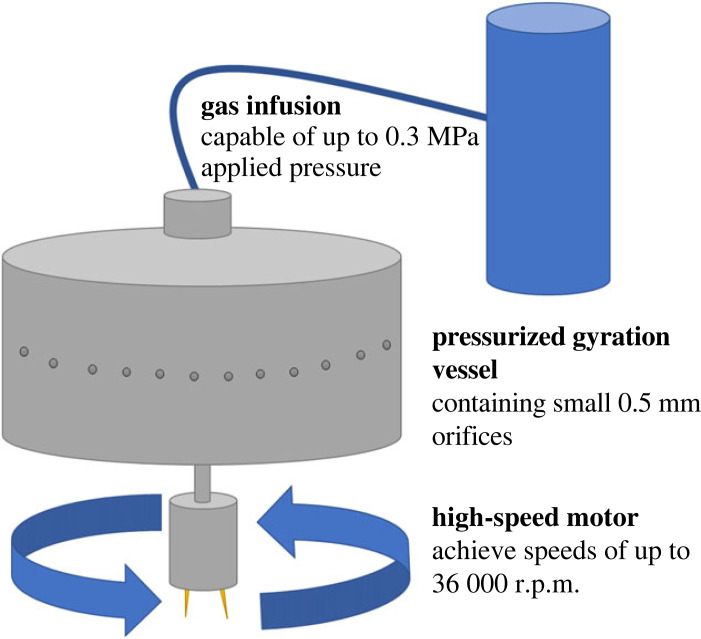
Diagram highlighting the main operating parameters of the pressurized gyration setup; including applied gas pressure, main gyration vessel and high-speed motor.

The casein–PCL polymer solution was spun with pressurized gyration, the basic setup of which is presented in figure 1. 2 ml of the polymer solution was placed in the central vessel, the speed was kept at a maximum rotational speed of 36 000 rpm and 0.1 MPa of applied pressure was added to aid with the fibre production process using nitrogen gas. An applied pressure of 0.1 MPa in the gyration vessel was determined to be optimal, as it would bring the highest yield and the most favourable fibre morphology [28,29]. Following spinning, the fibres were collected, and tests were subsequently carried out on them.

### 2.3. Cell viability assay

L929 (mouse fibroblast) cells were cultured in Dulbecco's Modified Eagle Medium (DMEM), supplemented with 10% fetal bovine serum (FBS) and then seeded in 48-well plates at a density of 2.5 × 10^4^ cells well^−1^ to be cultured overnight at 37°C and 5% CO_2_. Cells were treated with casein-loaded PCL fibres (CLF) (at 1 mg ml^−1^ concentration) and pure PCL fibres (PF) (at same sample size as CLF) for 48 h. 20% DMSO (positive control) and PBS were preferred as the positive and negative control groups, respectively [35]. After 4 h, formazan crystals were solubilized by adding 200 μl of solubilization buffer and the spectrophotometrical absorbance was measured at 550 and 690 nm. The confluent cells were used in the cytotoxicity tests and scanning electron microscopy (SEM) investigations [36].

### 2.4. *In vivo* wound healing experiments

Permission for the *in vivo* experiments was granted by the Animal Experiments Local Ethics Committee at Marmara University (approval number: 27.2022mar). For each experiment, 32 adult Sprague-Dawley rats (male and female) of mass 300–350 g were obtained from Marmara University Experimental Animal Implementation and Research Center. The rats were housed in regulated rooms with controlled conditions: temperature (20–23°C), humidity (40–60%), and light (12 h light/dark regime). The animals were kept on a standard rodent pellet diet with tap water available ad libitum.

### 2.5. Experimental design of wound healing experiments

To induce experimental type-1 diabetes mellitus (T1DM) in the rats, streptozotocin (STZ), which was prepared in a sodium citrate buffer (0.1 mol l^−1^, pH 4.5), was administered by a single intraperitoneal injection of 60 mg kg^−1^ [37]. The rats were then left overnight without food, in a fasting state.

The diabetic status was confirmed 72 h after STZ injections by measuring the blood glucose level collected from a vein in the tail with blood glucose strips (Contour^TM^ PLUS, Bayer Diagnostics). The rats that had a blood glucose level higher than 300 mg dl^−1^ were classed as diabetic and included in the experiment. Wound formation studies were carried out after 10 days of STZ administration. The rats were kept for 10 days in a chronic hyperglycaemic state before experiments began [38].

The dorsal hair of diabetic rats was completely shaved and the skin was disinfected with povidone-iodine after anaesthetic administration by intraperitoneal injection of 25 mg kg^−1^ ketamine hydrochloride (Ketalar) and 10 mg kg^−1^ xylazine hydrochloride (Rompun). A sterile biopsy skin punch with a diameter of 8.0 mm (Kai Medical, BP-80F, Japan) was used to create full-thickness wounds, from the middle back of each rat to the deep fascia, according to the template [39]. The rats were divided into 3 groups, each consisting of 12 rats: 1. Control group (no treatment), 2. PF group (treated with fibre without casein), 3. CLF group (treated with only CLF). During the healing process, no dressings were changed and no other topical medication was applied. The rats were kept in pairs following the surgical incisions. To observe the changes to the size of the wounds, wound margins were analysed and photographed on days 0, 3, 7, 10 and 14 after the surgical incision by using 3 rats for each group at each time point. Originally, casein was considered as the sole material for this study, but would not form fibres on its own; therefore a high casein loading of 50 wt% within PCL fibres was chosen to maximize the casein content. The original area (day 0 area) was characterized as the zone covering the immediately wounded environment. Wound closure is expressed as percentage closure relative to the original size and was calculated according to equation (2.1) [40]:

2.1 $$\begin{array}{rl} & \text{\%}\;\mathrm{Wound}\;\mathrm{closure}\;\\ & \;=\;100\;\times \;\frac{\mathrm{Initial}\;\mathrm{wound}\;\mathrm{area}-N\mathrm{th}\;\mathrm{day}\;\mathrm{wound}\;\mathrm{area}}{\mathrm{Initial}\;\mathrm{wound}\;\mathrm{area}}. \end{array}$$

This equation was used to express wound closure. *N* varies between 0 and 14 days.

The body weight of rats was stringently monitored for all the tested groups. The blood obtained from the rat tails was used to monitor glucose levels using blood glucose strips. Insulin glargine therapy was administered in cases where weight loss was apparent and/or displaying high glucose levels (Sanofi-Aventis, Frankfurt, Germany) [39]. The rats were sacrificed on days 3, 7 and 14, and the entire wound area was collected for further analysis. The tissues were embedded in a 10% formalin solution for histopathology. Additionally, the removed wounded area was stored at −80°C for biochemical studies on the 14th day.

### 2.6. Implantation of fibrous bandages on rats

The produced fibres resembled wound healing bandages and thus were used to cover the wounds. During testing, the wounds were covered completely (diameter of 8 mm) by the fibres in order to analyse the therapeutic effects of each sample on diabetic wound healing. Following the applications of the fibres, the wounds were wrapped with 3 M Tegaderm 1624w elastic bands (60 mm × 70 mm, Tegaderm^TM^, 3 M Health Care, Germany) in order to allow the fibres to remain on the wounds for the duration of the tests.

### 2.7. Histopathological analysis

For histological examination, skin samples were fixed in 10% neutral-buffered formalin for a minimum of 48 h. After fixation, the tissue samples were dehydrated in graded ethanol series (70, 90, 96 and 100%), cleared in xylene, embedded in paraffin and sliced into 5 μm sections. The stained sections from each rat and five similar areas in each section were analysed by experienced histologists that remained unaware of the experimental groupings. The sections were then stained with hematoxylin and eosin (H&E) and Masson's trichrome for optical microscopic examination to assess skin injury. The sections were then examined and photographed with an optical microscope (LEICA DM 1000). Each visual field was scored according to reepithelialization and granulation tissue formation and measured the epithelial tongue length and wound epithelial areas from at least five animals, for each individual treatment [41,42]. Each collagen distribution was analysed with Masson's trichrome staining.

### 2.8. Biochemical analysis

The tissue samples containing the wounded areas were collected after decapitation. The wound sections were homogenized, and the supernatant was analysed using ELISA kits purchased from Bioassay Technology Laboratory, Korean Biotech Co. Ltd (Shanghai, China). Serum levels of TNF-α, TGF-β, IL-1β, NF-κB and IL-6 were subsequently detected by ELISA. All procedures were carried out carefully according to the manufacturer's instructions.

## 3. Results and discussion

**Figure 2.**
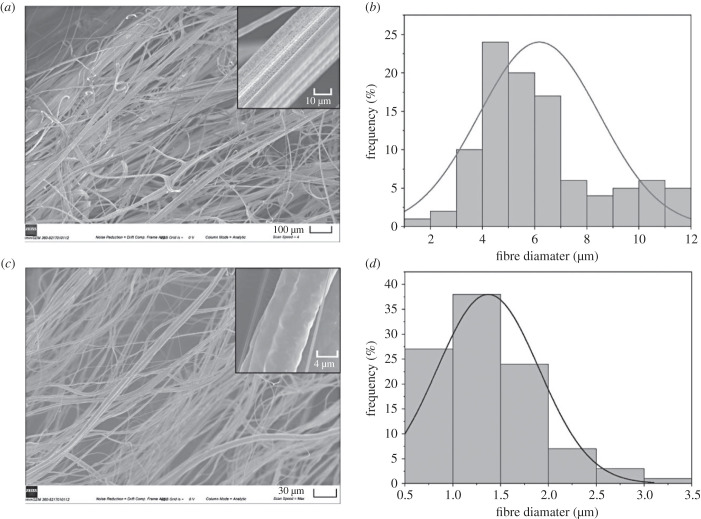
Scanning electron micrographs of: (*a*) virgin PCL fibres produced by pressurized gyration, including an inset to show fibre surface, and (*c*) casein-loaded PCL fibres including an inset to show fibre surface. (*b*,*d*) Respective graphs showing fibre diameter distribution, where *n* = 100 fibres.

Delivering the casein in the form of fibres creates one of the most efficient forms as a wound healing material. The fibrous bandage-like structure offers protection to the wound healing environment, while also providing the necessary porosity for the free flow of exudate. PCL was chosen as the polymeric support as it has great flexibility, toughness and biodegradability [43]. Figure 2 shows the scanning electron micrographs of the produced PCL and casein-loaded PCL fibres. Virgin PCL fibres, without the loading of any casein, displayed an average fibre diameter of about 6.2 ± 2.3 µm, with defined continuous fibre strands. The virgin PCL fibres do have some unidirectional alignment which can support cellular growth, as well as providing porosity for the wound healing process. Upon closer inspection of the fibre surface, it appears that the virgin PCL fibres also contain surface nanopores, a feature which can serve to increase the available surface area to volume ratio [44].

The loading of casein within the PCL polymer solution causes notable differences to the final fibre creation. In this work, a casein content of 50% was achieved in order to maximize its bioactive abilities. As casein is highly abundant, it does not become an economic limitation. The average fibre diameter for the casein-loaded PCL fibres was about 1.4 ± 0.5 µm, significantly lower than just using virgin PCL alone. The reduction in fibre diameter can be attributed to the presence of thicker casein particles within the polymer solution, causing a narrowing of the pressurized gyration vessels, leading to a smaller effective orifice size. The thinner casein fibres also displayed some unidirectional alignment with interconnected porosity. Upon closer analysis of the topography, the fibre surface appeared to have surface undulations and not nanopores. The addition of a high casein loading to the PCL fibres seems to reduce the porosity in the produced fibres, as it dilutes the level of solvent within the polymer solution.

### 3.1. Cell viability assay

**Figure 3.**
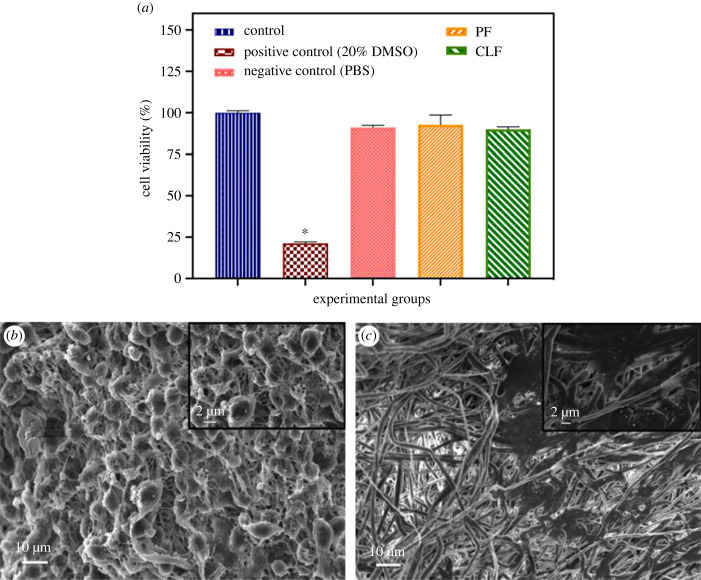
(*a*) L929 (mouse fibroblast) cell viability of all samples. SEM images of proliferated cells for (*b*) pure PCL fibre (PF) group and (*c*) casein-loaded PCL fibre (CLF) group after 48 h.

The evaluation of cytotoxicity was performed to determine the effects of the fibres on cell viability by using the MTT assay. According to the results, neither PF (92.8 ± 5.8%) nor CLF (90.1 ± 1.3%) had a cytotoxic effect on healthy mouse fibroblast cells compared to the control at 48 h (figure 3*a*). In addition, it was clearly seen that the cell clusters are correctly proliferating on all fibre samples through SEM analysis. It has therefore been proven that the fibres provide a suitable area for the growth of fibroblast cells (figure 1*b–d*) and the MTT test results of the fibres are backed up by the data shown in the SEM images. Consequently, these fibres can be safely recommended for further biocompatibility and animal testing and have great potential in biomedical applications due to their good cytocompatibility features. The results here can also be corroborated with those of many studies which show that casein had no toxicity on keratinocytes or any other cells of the wound healing response [45–47].

### 3.2. Evaluation of *in vivo* wound healing effect

**Figure 4.**
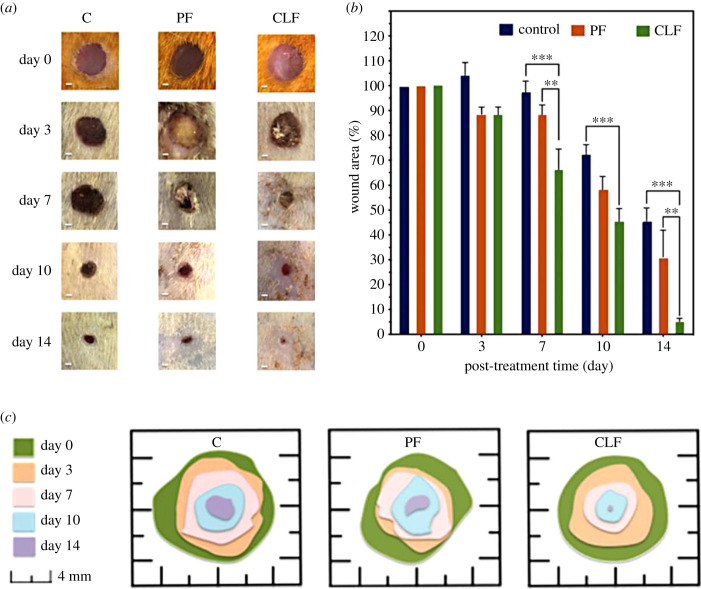
(*a*) Appearance of healing wound on days 0, 3, 7, 10 and 14 after surgical incision: (C) control group, (PF) pure PCL fibre group, and (CLF) casein-loaded PCL fibre group (scale bar = 1 mm). (*b*) Change in wound areas after various treatments. (*c*) Schematic representation of wound healing during the experiment. The data are presented as mean ± standard error of the mean. Significant differences were found at **p < 0.01, ***p < 0.001.

The wound model in STZ-induced T1DM rats was performed in this study to observe the wound-healing effects of the fibres. For this reason, the wound closure in diabetic rats was monitored for 14 days by taking photos on days 0, 3, 7, 10 and 14. The wound closure sizes of the diabetic rats were measured from day 0 to day 14 and expressed as percentages during the wound healing process; this can be seen in figure 4.

The activation of macrophages, endothelial cells, and fibroblasts generates a cellular response in wound healing. A typical biological process of wound healing in the human body consists of four different phases: haemostasis, inflammation, proliferation, and remodelling. In order for proper wound healing to commence, all four of these stages must be formed in a suitable sequence and time frame [48–51]. However, various physiological factors, namely diminished angiogenesis, persistent inflammatory response, and decreased fibroblast proliferation, can cause diabetic wound healing impairment [52,53].

Following the administration of PF and CLF, the evaluation of diabetic wound healing ability and change in wound area is elucidated in figure 4. On day 3, there is no significant difference in wound closure between the treated rats with PF and CLF (88.5 ± 2.8% and 88.2 ± 3.2%, respectively); however, these groups had mildly visible wound closures compared to the C group (103.7 ± 5.6%). An expressive difference in wound areas was observed in rats treated with CLF on days 7 and 10 by decreasing wound area values to 65.9 ± 8.6% and 44.5 ± 6.2%, respectively. The mean values of the wound areas in rats in the C and PF groups decreased to 45.6 ± 5.7% and 31.1 ± 10.8% while the wound treated with CLF was almost fully healed, and decreased to 5.2 ± 1.3% on day 14.

PCL, which has been approved by the FDA, is a biodegradable, hydrophobic polymer, and it degrades to naturally occurring metabolite 6-hydroxyhexanoic acid. Fibrous materials produced using this polymer support gas exchange and can regulate the humidity for the wound healing process. Thus, PCL mimics the native structure of the extracellular matrix and can mimic some skeletal constituents, due to its similar mechanical properties and biocompatibility [54,55].

Casein is a protein that composes 80% of cow's milk. It shows antimicrobial effects via the destruction of bacterial cell walls [56]. Furthermore, casein provides the formation of hydrogen bonds due to having polar amino acid groups. The combination of casein with the fibres can exemplify these properties and can make the delivery much simpler. In addition, casein supports the acceleration of haemostatic activity due to thrombin generation. Therefore, casein enhances epithelial proliferation by repairing the epithelial barrier integrity of wounds [57,58]. Based on our results, the most ideal wound healing conditions were obtained with CLF, while the PF group exhibited better results compared to the control group. On the 14th day, we can see that the wound is almost completely reepithelialized and significantly more covered than the control group wound.

β-Casofensin, a bioactive peptide derived from bovine casein, has also been shown to have pro-wound healing effects when given to rats. Rats which received β-casofensin showed improved levels of epithelial restitution [59]. In a different study using another casein-derived peptide called kashk, *in vitro* scratch test studies showed accelerated wound closure of about 44% when compared to the control group, due to the presence of kashk.

### 3.3. Histology

**Figure 5.**
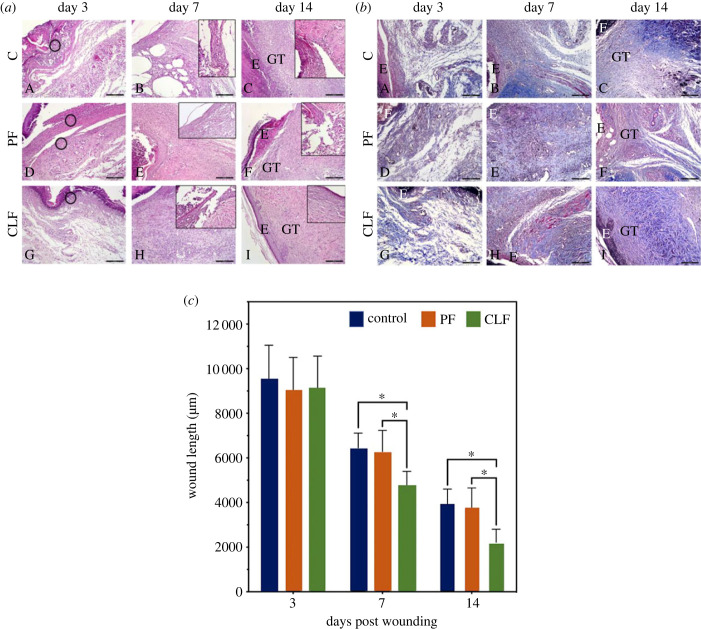
Histopathological evaluations. (*a*) General morphology and (*b*) collagen distribution of all groups, and (c) comparison of wound length between control, pure PCL fibres (PF), and casein-loaded PCL fibres (CLF) groups on days 3, 7 and 14. The circles indicate neutrophil infiltration. GT, granulation tissue; E, epithelium. Hematoxylin and eosin staining. Scale bars: 200 µm. Significant difference was found at *p < 0.05.

Results showing the gross appearances of the histology are shown in figure 5*a* (general morphology) and figure 5*b* (collagen distribution). The criteria used to evaluate the histological scores for wound healing are given in table 1 and the results of the histological evaluation are provided in table 2. Severe epidermis/dermis degeneration, neutrophil infiltration with increased bleeding, and edema were observed. However, no fibroblasts were detected in any of the groups on day 3. For the C and PF groups on day 7, neutrophil infiltration, altered angiogenesis and edema were more prevalent than in the CLF groups. The moderate epidermal and dermal organization were distinct in the CLF group compared to the other groups. Increased development of granulation tissue in the subepidermal region was observed in the CLF group compared to the C and PF groups. In all the tested groups, the wound width managed to decrease in contrast with day 3. On day 14, collagen distribution was mainly observed in all groups, while no bleeding was seen for any group. Almost complete reepithelialization and a high collagen density of the wound sites in the CLF group were observed, this being more prevalent than with the C and PF groups. Although neutrophil infiltration and edema were rarely seen in the C and PF groups, these findings were not observed in the CLF group. However, the formation of rat hair follicles was observed only in the CLF group on day 14, indicating a more complete wound healing.

**Table 1.** Criteria to evaluate histological scores of the wound healing.

score	epidermal and dermal regeneration	fibroblast density	neutrophil infiltration	edema
1	little epidermal and dermal organization	mild fibroblast density	mild	mild
2	moderate epidermal and dermal organization	moderate fibroblast density	moderate	moderate
3	complete remodelling of epidermis and dermis	increased fibroblast density	severe	severe

**Table 2.** Histological scoring on days 3, 7 and 14. H&E stained sections (*n* = 6 in each group) were scored as none (−), mild (+), moderate (++) and severe (+++).

groups/days	epidermal degeneration	fibroblast proliferation	neutrophils	edema
*(a) day 3*				
C	+++	−	+++	+++
PF	+++	−	+++	+++
CLF	+ +	−	+++	+ +
*(b) day 7*				
C	+++	+	+++	+++
PF	+ +	+	+ +	+ +
CLF	+	+ +	+	+
*(c) day 14*				
C	+ +	+	++	+ +
PF	+	+ +	+	+
CLF	−	+++	−	−

### 3.4. Biochemical analysis

Proinflammatory cytokines, including tumour necrosis factor α (TNF-α), transforming growth factor β (TGF-β), interleukin (IL)-1β, and IL-6, exhibit a significant role as the primary factors produced in the wound healing process. They organize the immune cell functions, and their overexpression can cause prolonged inflammation via the activation of downstream cascades. On the other hand, they promote cell proliferation, keratinocyte antimicrobial peptide production, and differentiation by mobilizing resident stem/progenitor cells. TNF-α promotes the cell surface adhesion molecule synthesis for adhesion to the endothelium and neutrophil migration. IL-1, which is produced by neutrophils, keratinocytes and macrophages, promotes the secretion of IL-6 from fibroblasts, keratinocyte growth factor, granulocyte-macrophage colony-stimulating factor, fibroblast growth factor-7, and hepatocyte growth factor. Therefore, a paracrine loop in the wound-healing process is created through the bidirectional interactions between fibroblasts and keratinocytes. IL-6 induces skin fibroblast activation and expresses pro-regenerative factors [60,61]. Additionally, it increases TGF-β expression. TGF-β affects various processes in wound healing such as collagen synthesis, inflammation, fibroblast proliferation, stimulation of angiogenesis, deposition and remodelling of the new extracellular matrix [62]. Nuclear factor-kappa B (NF-κB), which is a ubiquitous transcription factor, has important roles such as in the formation of immune responses and inflammation, and the regulation of expression of many other genes involved in differentiation, cell survival, and proliferation [39,63].

**Figure 6.**
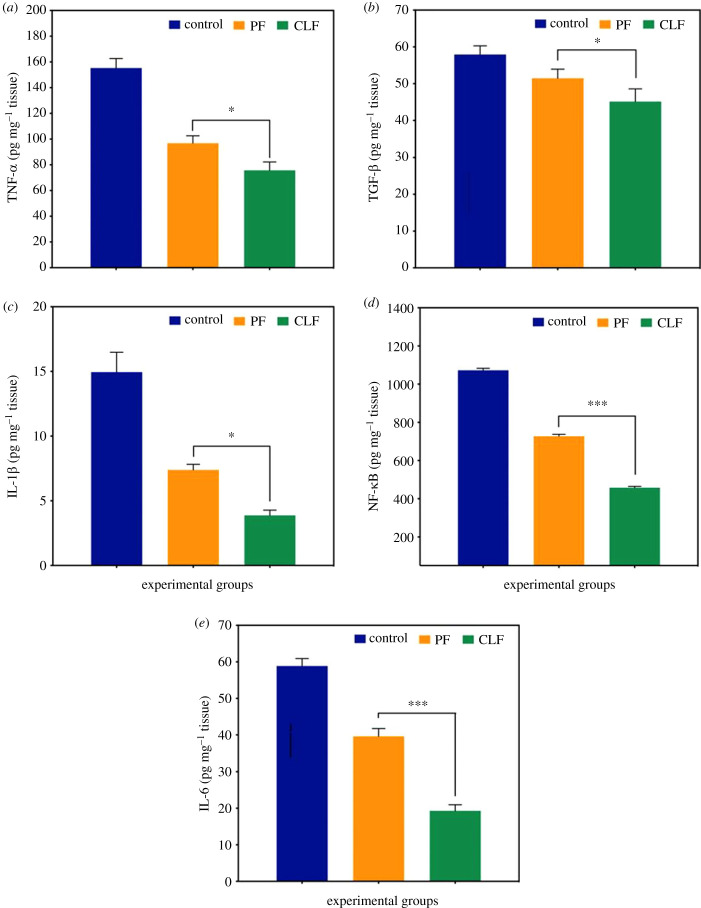
The levels of TNF-α, TGF-β, IL-1β, NF-κB, and IL-6 in the wounded area on day 14. The data are presented as mean ± standard error of the mean. *p < 0.05 and ***p < 0.001.

The levels of TNF-α, TGF-β, IL-1β, NF-κB and IL-6 in the CLF group were found to be lower compared to the C group in this study (figure 6). While the levels of NF-κB were detected at 1073 and 456 ng mg^−1^, the levels of TNF-α were detected at 155 and 76 pg mg^−1^ in the C and CLF groups, respectively. Notably, there were significant differences in the levels of NF-κB and IL-6 between each group (*p* < 0.001). The values for the PF group were found to be more suitable when compared with group C, while the CLF group exhibited the largest decrease in cytokine levels. There were no significant differences observed between the C and PF groups in the level of IL-1β. These results proved that casein has an important role in the wound healing process by decreasing the levels of TNF-α, TGF-β, IL-1β and NF-κB.

The results show a significant difference between the virgin PCL fibres and the casein-loaded fibres, strongly indicating that casein does indeed exhibit a bioactive response which regulates the overexpression of TNF-α, TGF-β, IL-1β, NF-κB and IL-6. By combining all the data collected in this work, it becomes apparent that casein is a viable means of increasing the wound healing performance of fibrous constructs using a natural and sustainable material addition.

## 4. Conclusion

Small-diameter fibrous bandage-like mats were successfully produced using a high loading of casein within PCL. The addition of casein into the fibres improved their morphology and their potential application as a wound healing scaffold. The casein-loaded fibres showed good unidirectional alignment and had an average fibre diameter of 1.4 ± 0.5 µm. Fibroblast viability studies showed that there was no significant cytotoxicity in any of the tested samples. Casein-loaded fibres showed the greatest reduction in wound area following treatment, where almost complete regeneration was seen in the 14-day testing period. Histological evaluation revealed increased levels of granulation tissue development for the casein-loaded bandage-like fibres and the reformation of hair follicles on the wounded site. The levels of TNF-α, TGF-β, IL-1β, NF-κB and IL-6 were all significantly lower for the groups that were given casein-loaded fibres compared to the control group and the virgin PCL fibres. This work demonstrates the effectiveness of casein, a highly abundant and inexpensive protein which can be used in wound healing materials, to improve the healing process. Furthermore, casein has the potential to provide antibacterial protection as well, which should be explored by further studies.

## Data Availability

This work does not contain supplementary material, but data can be provided on request.
